# Medical empathy in medical students in Madrid: A proposal for empathy level cut-off points for Spain

**DOI:** 10.1371/journal.pone.0267172

**Published:** 2022-05-23

**Authors:** José Manuel Blanco Canseco, Augusto Blanco Alfonso, Fernando Caballero Martínez, María Magdalena Hawkins Solís, Teresa Fernández Agulló, Lourdes Lledó García, Antonio López Román, Antonio Piñas Mesa, Elena Maria Vara Ameigeiras, Diana Monge Martín

**Affiliations:** 1 School of Medicine, Universidad Francisco de Vitoria, Pozuelo de Alarcón, Madrid, Spain; 2 Valle de la Oliva Healthcare Centre, Majadahonda, Madrid, Spain; 3 Reina Victoria Healthcare Centre, School of Medicine, Universidad Autónoma de Madrid, Madrid, Spain; 4 Dean School of Medicine, Universidad Francisco de Vitoria, Pozuelo de Alarcón, Madrid, Spain; 5 School of Biomedical and Health Sciences, Universidad Europea de Madrid, Madrid, Spain; 6 Faculty of Health Sciences, Universidad Rey Juan Carlos, Madrid, Spain; 7 Dean Faculty of Medicine and Health Sciences, Universidad de Alcalá, Madrid, Spain; 8 School of Medicine, Universidad Alfonso X el Sabio, Madrid, Spain; 9 School of Medicine, Universidad CEU San Pablo, Madrid, Spain; 10 School of Medicine, Universidad Complutense de Madrid, Madrid, Spain; University of Eastern Finland: Ita-Suomen yliopisto, FINLAND

## Abstract

This study evaluates the degree of empathy among medical students and its influencing factors at three critical moments of their degree studies (beginning of first year and end of third and sixth years) as well as establishes low-, medium-, and high-empathy cut-off points to obtain valid and reliable results that can be extrapolated to the general population. This cross-sectional study of the eight (public and private) medical schools in the province of Madrid, used an electronic questionnaire with the Jefferson Scale of Empathy (JSE), Medical Student Well-Being Index, and other independent characteristics as measuring instruments. Of the 2,264 student participants, 1,679 (74.0%) were women, with a 50.7% participation rate. No significant differences were found in empathy levels by academic year. Regarding range, percentile and cut-off point tables were established to identify students with high, medium, and low empathy levels. Women (p<0.001), volunteer workers (p<0.001), and those preferring general specialties (internal medicine, psychiatry, pediatrics, or family medicine) scored higher on the JSE (p<0.02). Moreover, 41.6% presented high level of psychological distress. Women reported a lower well-being level and a higher risk of psychological distress (p = 0.004). In sum, the empathy of medical students in Madrid did not differ among the three critical moments of their university studies. The established cut-off points could be taken into account when accessing the medical degree and identifying students with low levels of empathy to implement curricular interventions to rectify this perceived deficiency. There was a high percentage of medical students with high levels of psychological distress.

## Introduction

Empathy is a personal quality that involves feeling, imagining, and understanding the inner world of others [1]. Empathy is a multidimensional construct comprising cognitive, affective and behavioural dimensions [2, 3]. Medical empathy is a fundamentally cognitive ability [2, 4] which also involves knowing how to transmit this understanding to the patient with the intention of helping to prevent or alleviate their suffering [4, 5].

The communicative and empathic skills of physicians are key to their relationships with their patients and are considered a core element of professionalism [6]. Organizations such as the International College of Person Centered Medicine (http://www.personcenteredmedicine.org/) [7] and the European Society of Person Centered Healthcare (http://www.pchealthcare.org.uk/) [8] advocate the need to reinforce these skills to improve the health of both, people who are sick and their caregivers.

Greater empathy in doctors is associated with greater patient satisfaction [9], stronger adherence to treatments [10] and more favorable health outcomes [11, 12]. In professionals, it is associated with more effective clinical skills [13] and lower burnout rates [14]. More empathic medical students have greater well-being [15], less burnout [16, 17] and better clinical competence [18–20].

Empathy can be improved with correct training [21–23]. Medical schools play a key role in evaluating and monitoring the empathic skills of their students and in carrying out educational actions that maintain and maximize them [24]. Some authors [4, 24, 25] argue that admissions to universities should consider the humanistic qualities of applicants who are to be future doctors, in addition to their academic qualifications. Some countries already incorporate professional aptitude tests in access to the Degree in Medicine in order to select students with a profile of more humanistic values such as empathy, communication, compassion or social commitment [26].

The Jefferson Scale of Empathy (JSE) [27] is a valid and reliable instrument developed specifically to measure the degree of empathy among medical students and practicing physicians. It has been translated and culturally adapted into more than 56 languages/dialects and has been employed in at least 85 countries. Currently, it is the most widely used tool to measure empathy in medical education [2]. In Spain, various studies have shown the validity and reliability of the JSE [28–31].

Most of the studies in our field [28, 30–32] which analyze variables associated with higher or lower levels of empathy in medical students have been performed in a single institution or with small, unrepresentative sample sizes. Consequently, any generalization of these results could be questioned. It is therefore unclear how medical students’ empathy evolves over time, with different studies reporting conflicting results. Some studies [33–36] show a clear fall in empathy throughout the degree course, especially when students come into contact with patients in their clerkships, while this trend is not apparent in other studies [37–40]. In a recent systematic review, Ferreira-Valente et al. [39] report a significant deterioration in the level of empathy among medical students when analyzing different longitudinal studies, whereas cross-sectional studies did not corroborate this trend. Other longitudinal studies carried out in this setting observe that empathy does not deteriorate over time [31].

Different factors may affect the levels of medical students’ empathy throughout their degree studies. These include cultural differences, characteristics of the healthcare systems to which they belong, workload, burnout, emotional overload, and lack of good mentors [34, 35]. Studies have found that students in the final years of their education have a high prevalence of depression and anxiety, and that levels of psychological distress are higher than those of the general population and their age-matched peers. Taken together, the studies suggest that psychological distress may be higher among female students [41–43]. A study conducted in all 43 medical Spanish schools shows similar results [44]. Lower levels of empathy are associated with greater burnout and a lower climate of professionalism [45]. Students with signs of depression show lower empathy [44]. More empathic medical students have greater well-being [15] and less burnout [16, 17]. There is thus a growing need to analyze medical students’ empathy in large samples that represent their populations of origin, thereby making the establishment of percentile tables and cut-off points at a national level possible [46]. Having these data will allow us to detect higher and lower levels of empathy in students and thus assess the impact of different curricular activities that seek to reinforce and enhance it. Hojat and Gonella (2015) and Hojat et al. (2018) [46, 47] already achieve this with medical and osteopathy students in the United States.

The present study aims to evaluate the degree of empathy in a large sample of students from the eight medical schools in the province of Madrid at three critical moments in their degree studies (first, third, and sixth academic years). Given that these eight medical schools represent 20% of the 42 medical schools in the country and receives students from all over the national terrritoriy, this work has the following objectives: it aims to establish rank-percentile tables and cut-off points to identify students with high, medium, and low levels of empathy in Spain. Moreover, it seeks to deepen—with a higher level of reliability and internal and external validities than those of previous studies—the knowledge of different variables that could affect the level of empathy in medical students. These variables include their preferences for a particular specialty, current academic year, professional experience, involvement in volunteer work, or previous experience of serious illness in themselves or family members. It is of particular interest to know the degree of the well-being of medical students and its influence on their levels of empathy.

The present research is an initial study within a project that seeks to evaluate the Empathy of Medical students in Madrid over time (Project EMMA) and identify the determinants of student empathy and analyze the effect that different curricular activities may have on it.

## Materials and methods

### Design

This is a descriptive cross-sectional study conducted at three critical moments during the degree course in medicine: beginning of the first year and end of the third and sixth years. Data were collected in May and September 2019 prior to the COVID-19 pandemic. The work was coordinated by a research committee in which at least one professor coordinator of each medical school was represented. It had the approval and support of the deans of the eight participating medical schools.

### Scope and participants

Participants included 2,264 first-third-sixth-year students (74.0% women, n = 1,679) from 8 medical schools in Madrid, Spain (out of 8), four of which were public and four were private, representing 100% of all schools of medicine. All students in each course were invited to participate by consecutive sampling.

### Measuring instruments

Students responded to an electronic, anonymous, and voluntary questionnaire. The faculty of medicine managers and lecturers explained the purpose of the study to the students. Subsequently, after giving their consent to participate, the students were given time at the beginning or end of one of the classes to complete the questionnaire. As a dependent variable, self-perceived empathy by medical students was measured using the JSE. The JSE’s Spanish translated and validated version for healthcare professionals (JSE-HP) was used for medical students from Madrid [30]. This scale was specifically designed to assess empathy in the physician-patient relationship from a more cognitive point of view, and it is the most used instrument globally to evaluate the empathy of medical students [2, 27]. The JSE-HP can be used to assess the empathy of medical students who have already had contact with real or simulated patients (usually from the third year onwards) [5, 48]. First-year students who had not yet been in contact with patients were asked to make an effort to imagine the experience for themselves, rather than imagining what the situation might be like for another person. Hojat et al. [49] find no differences between the generic version for students and the physician-specific version in a before-after cross-over study with 42 internal medicine residents. The correlations between the scores of the two versions were 0.85 (p<0.01), with no differences in Cronbach’s alpha or significant changes in scale scores.

The JSE-HP consists of 20 items with a seven-point Likert scale (1 = strongly disagree to 7 = strongly agree). Ten of the 20 issues are rated negatively (with positive rectification in the subsequent analysis) to reduce the effect of the acquiescence response bias. The possible scores ranged from 20 to 140 points, with higher scores associated with a higher degree of empathy. Although there is no time limit, the questions are usually answered in less than five minutes. The scale is considered valid if at least 80% of the questions are answered. If more than four questions remained unanswered, the questionnaire is considered null and void [4, 48].

In the original version and subsequent studies, factorial analysis [5] revealed three dimensions of the scale. Dimension 1: The patient’s perspective (cognitive aspects of empathy) consisting of 10 items; Dimension 2: Attention with compassion (emotional aspects of empathy) consisting of 8 items; and Dimension 3: Putting yourself in the place of the patient, consisting of 2 items.

The cut-off points of low and high empathy were defined, as described by the authors [46] of the scale, by arbitrarily determining two points in the score distribution. To identify high levels of empathy, a cut-off point of one and a half standard deviation above the mean score was chosen. To identify low levels of empathy, one and half standard deviation below the mean score was chosen.

The following independent variable predictors of empathy were analyzed: age, gender, academic year, nationality, coexistence in the family nucleus, compatibility of studies and work, previous professional experience, experience of illness (one’s own or of a close family member), preference by type of speciality desired for future residency, and participation in volunteer work. Students were asked to choose one of the following four groups of specialties: general specialties (internal medicine, psychiatry, pediatrics, and family medicine), other medical specialties (cardiology, digestive, nephrology…), surgical and technological specialties (surgeries, radiodiagnosis, etc.), and other non-clinical specialties (clinical analysis, pathological anatomy, and preventive medicine). These types of groupings have been used in previous studies [4, 5, 50].

The level of the students’ well-being and psychological distress was analyzed using the Medical Student Well-Being Index (MSWBI) [51, 52] of the Mayo Clinic. This questionnaire has seven items [4]. In its construct, psychological distress encompasses depression, anxiety, burnout, and related mental health problems. The overall score is the sum of these seven items, and it ranges from 0 to 7 points. The higher the score, the higher the students’ degree of psychological distress and the lower their well-being. The cut-off point for detecting students with a high degree of psychological distress is four or more points [53].

### Statistical analysis

Qualitative variables were described using their frequency distributions and percentages. Quantitative variables were described with their mean and standard deviation (SD) when they followed a normal distribution pattern and with median and interquartile range (P25–75) for non-parametric distributions.

The association between qualitative variables was carried out using the chi-square test. The relationship between continuous variables and qualitative variables was determined by the t Student for independent samples or analysis of variance when the variables presented a normal distribution. The non-parametric Mann-Whitney U test or the Kruskal-Wallis test was employed when the variable was proven to be asymmetric. The association between continuous quantitative variables was determined using Pearson or Spearman correlations. In cases wherein it proved necessary, the effect’s magnitude was estimated when the association was statistically significant, using the Cohen’s d test, to determine the practical (clinical) importance of statistically significant results. Effect magnitudes smaller than 0.2 were not considered to be of importance [54]. A multiple linear regression model (with JSE as a dependent variable) was used to assess the independent effect of each of the other variables that could influence levels of empathy. We used gender (women vs. men), volunteer work, serious illness, work placements started, Spanish nationality, living with your family as binary variables, and preference by specialty as a categorical variable. Total MSWBI, age, and the academic year were continuous variables.

For statistical analysis, the IBM-SPSS Stadistics_21 program for Windows was used, with a priori significance level of alpha<0.05, in all analyses.

### Ethical aspects

To respect international data protection standards as well as the Spanish legislation in force—Organic Law 3/2018 of December 5 on the Protection of Personal Data and Digital Rights Guarantees, Official State Bulletin (BOE) 294, dated 6/12/2018—all questionnaires were anonymous. The Ethics Committee of the Universidad Francisco de Vitoria approved the study. Students’ participation was voluntary, the informed consent was written and their (non-)participation did not affect their academic results.

## Results

A total of 2,301 students completed the questionnaire. Of these, 37 questionnaires were discarded because students failed to answer more than four JSE items. All told, 2,264 of the 4,469 students enrolled in the first, third, and sixth years of the eight medical schools in Madrid participated in the study. The overall participation rate was 50.7%. Participation rates among the first-, third-, and sixth-year students were 59.7% (n = 893), 46.3% (n = 714), and 45.9% (n = 657), respectively. Their mean ages were 18.9 (SD = 2.8) years, 22.2 (SD = 2.0) years, and 25.1 (SD = 1.8) years, respectively. Further, the number of women participants from each year was 681 (76.3%), 536 (75.1%), and 460 (70.1%), respectively. Gender distribution in the study sample did not differ from that in the source population.

Of the students, 95.1% (n = 2,157) were Spanish nationals. A total of 62.9% (n = 1,426) reported living with their family, and 10.7% (n = 243) shared their studies with some form of work activity. Further, 34.2% (n = 775) had previous professional experience. In 15.8% of cases (n = 122), this professional experience was in the health field.

Of the sample studied, 33.1% (n = 750) reported having participated in volunteer work. This percentage was significantly higher in women (35.7%) than in men (25.3%; p<0.001). Moreover, 68.2% (n = 1,547) reported having had a previous experience of serious illness, be it their own or that of a close family member. This percentage was higher in men (38.3%) than in women (32.8%; p = 0.017).

Further, 31.7% of the students (n = 718) opted for a general specialty (internal medicine, psychiatry, pediatrics, or family medicine), 27.6% (n = 625) chose other medical specialities (cardiology, digestive, nephrology, etc.), 32.1% (n = 727) preferred surgical or technological specialities (surgeries, radiodiagnosis, etc.), and 8.7% (n = 198) chose non-clinical specialities (clinical analysis, pathological anatomy, or preventive medicine). By analyzing this preference by academic year, students in their sixth year, compared to those in their first, chose more general specialities (37% in the sixth vs. 27% in the first) and medical specialities (33.5% vs. 23.7%) compared with surgical or technological (25.7% vs. 33.3%) and non-clinical (3.8% vs. 16%; p<0.001) specialities.

Internal consistency of JSE-HP is shown in Table 1. No item is dispensable as the Cronbach’s alpha obtained of 0.80 would not significantly improve. Pearson correlation coefficients were calculated to examine correlations between each item score and the total score of the JSE. There is positive and significant correlation between each of the items and the overall result of the scale, the median being 0.39 (p< 0.01).

**Table 1:** Reliability of JSE-HP in its Spanish version applied to a national sample of 2,268 students at the beginning of academic year from 8 campuses of colleges of medicine in Spain.

Item	Mean	Standard deviation	Alpha if item eliminated	Corrected item-total score correlation
Item 1	6.09	1.93	0.80	0.24^a^
Item 2	6.64	0.84	0.79	0.37^a^
Item 3	5.55	1.48	0.79	0.34^a^
Item 4	6.23	1.16	0.79	0.31^a^
Item 5	5.21	1.49	0.80	0.16^a^
Item 6	5.97	1.22	0.78	0.41^a^
Item 7	6.23	1.37	0.79	0.37^a^
Item 8	6.30	1.33	0.79	0.42^a^
Item 9	6.17	1.19	0.78	0.50^a^
Item 10	6.15	1.13	0.78	0.53^a^
Item 11	6.15	1.29	0.78	0.48^a^
Item 12	5.78	1.89	0.80	0.29^a^
Item 13	6.04	1.21	0.78	0.50^a^
Item 14	6.27	1.45	0.79	0.35^a^
Item 15	6.27	1.29	0.79	0.45^a^
Item 16	6.29	1.03	0.78	0.61^a^
Item 17	5.75	1.33	0.78	0.45^a^
Item 18	3.83	1.54	0.80	0.17^a^
Item 19	6.13	1.53	0.80	0.21^a^
Item 20	6.53	0.91	0.78	0.56^a^

^a^ Correlations between scores on each item and the JSE total score by excluding the corresponding item from the total score. All correlations are statistically significant (*p* < 0.01)

The overall mean JSE score was 115.22 points (SD = 14.24) for men and 121.17 points (SD = 11.18) for women. Table 2 reflects the sample empathy levels with ranges and percentile ranges disaggregated by gender. Table 3 reflects the JSE scores (total and by dimensions) of the sample by academic year.

**Table 2:** Frequencies, percentage distribution, and descriptive statistics of Jefferson Scale of Empathy scores in 2,268 medical students in Madrid, disaggregated by gender (men, N = 588 [26.0%]; women, N = 1,679 [74.0%]; total, N = 2,268).

Interval	Men Freq.	Cumulative Freq.	Range Percentile %	Women Freq.	Cumulative Freq	Range Percentile %	Total Freq	Cumulative Freq.	Range Percentile %
≤80	12	12	1–2	4	4	1	16	16	1
81–85	8	20	3–4	9	13	1	17	33	1–2
86–90	12	32	5–6	15	28	2	27	60	3
91–95	26	58	7–11	24	52	3	50	110	4–5
96–100	23	81	12–15	31	83	4–5	54	164	6–8
101–105	43	124	16–22	48	131	6–8	91	255	9–12
106–110	56	180	23–33	123	254	9–16	179	434	13–20
111–115	69	249	34–45	176	430	17–27	245	679	21–32
116–120	85	334	46–61	212	642	28–40	297	976	33–46
121–125	75	409	62–74	296	938	41–59	371	1.347	47–63
126–130	68	477	75–86	306	1.244	60–79	375	1.722	64–81
131–135	57	534	87–97	238	1.482	80–94	295	2.017	82–95
>135	18	552	98–100	91	1.573	95–100	109	2.126	96–100
Losses	36	588		106	1.679		142	2.268	
Total		588			1.679			2.268	
Descriptive statistics									
Mean average	115.22			121.17			119.60		
Median	117			123			122		
S.D.	14.24			11.18			12.33		
Asymmetry	-0.95			-0.94			-1.05		
Kurtosis	0.21			1.08			1.95		
Possible range	20–140			20–140			20–140		
Current range	31–140			70–140			31–140		
Cronbach’s alpha	0.73			0.62			0.82		

**Table 3:** Jefferson Scale of Empathy (JSE) scores by academic year and gender from the sample of 2,268 medical students from Madrid.

Year		First		Third		Sixth	
Gender		Men	Women	Men	Women	Men	Women
Total JSE	Mean Average	114.69	120.44	112.72	121.28	117.81	122.08
	SD	13.41	13.38	15.35	11.73	13.61	10.14
Dimension 1	Mean Average	59.15	61.71	59.14	62.31	60.32	61.69
	SD	8.26	6.60	9.31	6.83	7.55	6.15
Dimension 2	Mean Average	44.54	47.04	43.16	47.25	46.30	48.54
	SD	7.10	6.88	8.50	6.58	6.49	7.00
Dimension 3	Mean Average	11.01	11.68	10.42	11.72	11.19	11.85
	SD	2.26	2.20	2.94	2.30	2.28	2.09

Note: SD = standard deviation.

**Fig 1:**
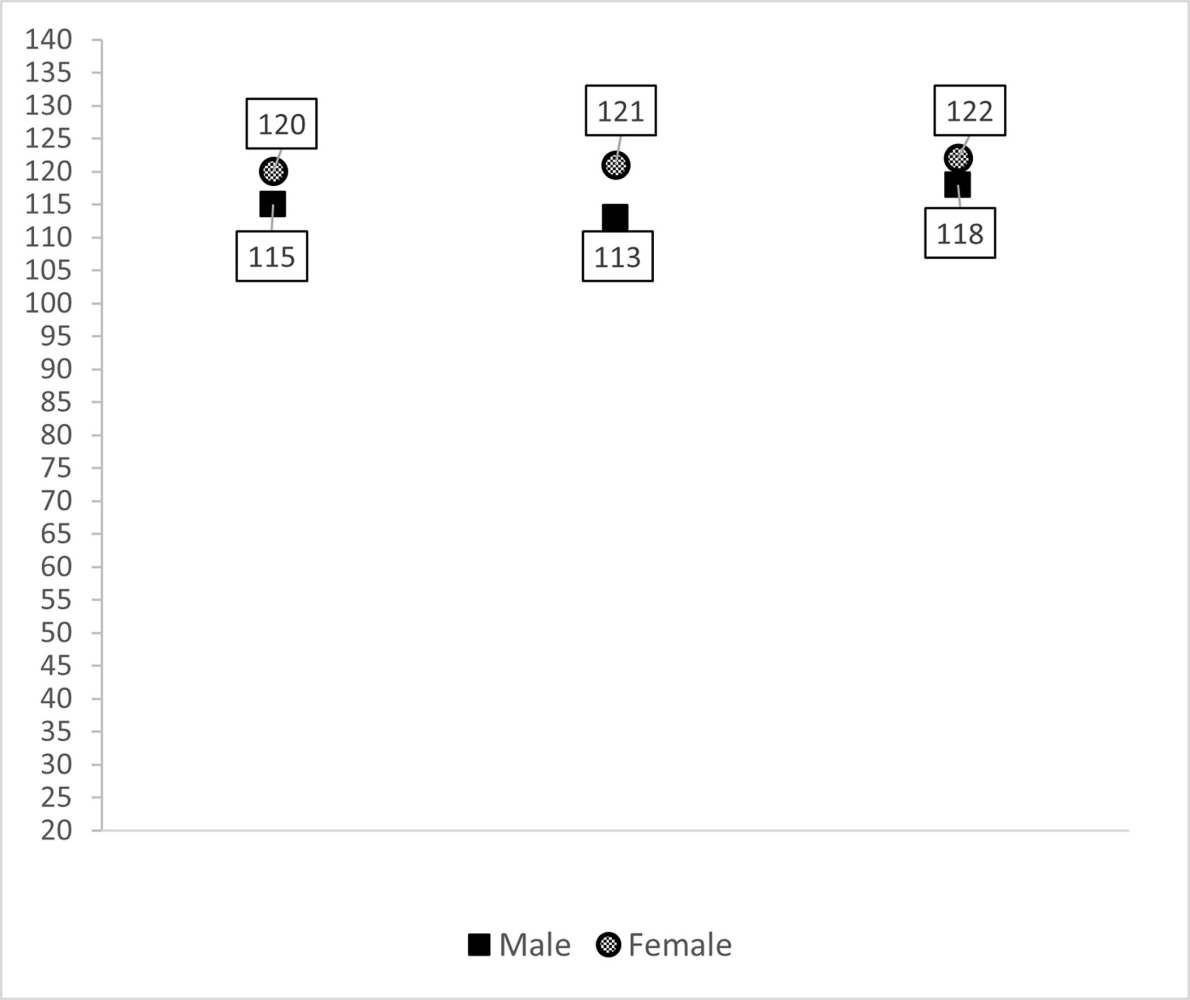
Overall Jefferson Scale of Empathy (JSE) scores based on the academic year and gender of 2,264 medical students.

In the analysis of variables that could influence student empathy, women scored a mean of 6.05 (95% confidence interval [CI] 4.73–7.36) points higher than men in overall JSE scores (p<0.001; Cohen’s d: 0.46). Fig 1 shows the overall total JSE scores disaggregated by gender across academic years.

There were no differences in levels of empathy among the first-, third-, and sixth-year students, except the third- and sixth-year male students. Sixth-year male students scored an average of 5.09 points more than those in their third year (95% CI 1.50–8.68 points; Cohen’s d = 0.35).

**Fig 2:**
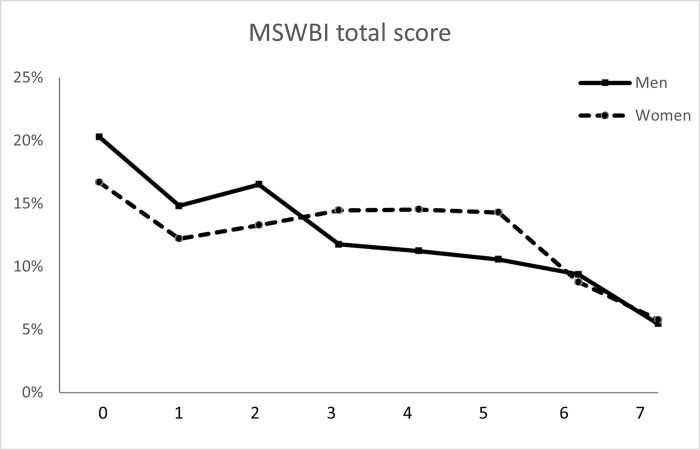
Medical Student Well-Being Index (MSWBI) frequency distribution by gender in 2,264 medical students.

The overall mean well-being (MSWBI) score was 2.76 points (SD = 2.19) for men and women had 3.05 points (SD = 2.13); the mean difference was 0.29 points (95% CI 0.09–0.50; p = 0.004; Cohen’s d = 0.13). In addition, 41.6% of the students (n = 944) scored 4 or higher (psychological distress). This percentage was significantly higher in women than in men (43.4% vs. 36.7%; p = 0.007; see Fig 2).

Students who preferred general specialities (internal medicine, psychiatry, pediatrics, or family medicine) scored a mean JSE score of 121.85 points (SD = 11.62). Those who chose other medical specialities (cardiology, digestive, nephrology, etc.) had 119.80 points (DE = 12.33) points, and those who chose surgical-technological (surgeries, radiodiagnosis, etc.) and non-clinical (clinical analysis, pathological anatomy, or preventive medicine) specialities scored 118.46 (SD = 12.11) and 114.86 (SD = 13.91) points, respectively. In the post-hoc tests of multiple Bonferroni comparisons, these differences were statistically significant for all subgroups, except when the scores of those who chose other medical specialities versus surgical-technological specialities were compared. These differences remained significant after controlling for age and gender (Tables 4 and 5).

**Table 4:** Comparison of average Jefferson Scale of Empathy scores based on preference for a particular specialty.

Specialties		Mean averages difference	p*	Confidence interval 95%	Cohen’s d
General specialties	Medical specialties	2.05	0.02*	0.23–3.86	0.17
	Technical-surgical specialties	3.39	0.00*	1.64–5.13	0.29
	Non-clinical specialties	6.99	0.00*	4.28–9.70	0.55
Medical specialties	Technical-surgical specialties	1.34	0.31	-0.47–3.14	0.11
	Non-clinical specialties	4.94	0.00*	2.19–7.69	0.38
Surgical-technological specialties	Non-clinical specialties	3.61	0.00*	0.90–6.31	0.28

**Table 5:** Linear regression model of Jefferson Scale of Empathy (JSE) scores and other variables.

Model	Coefficients	Sig.
	beta	
(Constant)		<0.001
Gender (woman/man)	-0.201	<0.001
Volunteer work (no/yes)	0.103	<0.001
Serious illness (no/yes)	0.035	0.091
Work placements Started (no/yes)	-0.005	0.914
Spanish nationality (no/yes)	0.042	0.05
Living with your family (no/yes)	0.041	0.057
Total MSWBI (the higher the MSWBI, the higher the distress)	-0.071	0.005
Preference by specialty (General/medical/surgical-technological/non-clinical)	-0.124	<0.001
Academic year	0.067	0.152
Age	0.026	0.440

Note: Dependent variable: total JSE; R^2^ corrected = 0.083

Students engaged in volunteer work obtained mean scores in the total JSE of 121.8 points (SD = 11.50) versus 118.5 points (SD = 12.59) for those who did not. Mean averages difference: 3.31 (95% CI 2.24–4.38) points (p = 0.001; d Cohen 0.13). 0.27). This difference remained significant after controlling for age and gender (Table 5).

The mean overall empathy score for students who had had a previous experience of serious illness, be it personally of that of a family member, was 118.85 points (SD = 12.92) compared with 119.95 points (SD = 12.04) for those who had no such experience (p = 0.056).

To analyze the independent effect of each variable on empathy, we performed a linear regression model (Table 5), controlled for age and gender. The characteristics associated with higher empathy were as follows: being a woman (β = -0.201, p<0.001), having performed some form of voluntary work (β = 0.103, p<0.001), reporting a higher level of well-being (β = -0.071, p<0.005) and reporting a preference for a general medical speciality (β = -0.124, p<0.001).

As in the present study and a large number of previous studies [4, 18, 46, 47], there are differences in empathy according to gender (higher values in women), and so we set different cut-off points for men and women. As no differences in empathy were found between academic years, the calculations were made with the total sample of students from the three years studied [50]. Table 6 shows the proposed empathy level cut-off points for Spanish medical students. Thus, the frequency distribution table and the cut-off points proposed for low, medium, and high empathy can be used in other Spanish universities, provided the students’ demographic characteristics and JSE scores do not differ substantially from those presented in Table 2.

**Table 6:** Proposal for cut-off points of empathy levels in Spain after analyzing Jefferson Scale of Empathy scores in 2,268 students in Madrid (EMMA).

Level	Gender	Hojat et al. [46]	EMMA
Low	Men	≤95	≤93.86
	Women	≤100	≤104.4
High	Men	≤127	>136.58
	Women	≥ 129	≥ 137.94

Note: Comparison with cut-off points presented by Hojat et al. [39] in 2,637 students at Sidney Kimmel Medical College.

## Discussion

The sample size of the study is much larger than that in previous research [28, 29, 31] analyzing empathy in this field. All of Madrid’s medical schools, both public and private, participated in the study. The province of Madrid has a population of 6,745,591 inhabitants, 14.2% of the Spanish population. 20% of the country’s medical schools are concentrated in this community, receiving students from all over the country. Consequently, the conclusions presented here may be considered highly reliable (consistency and repeatability) as well as sound regarding internal validity (true relationship between variables) and external validity (generalization).

The response rate to the questionnaire (50.7%) was higher than the usual rate in previous research employing online questionnaires. Cho et al. [55] report an average online questionnaire response rate of 35% in a review study. Hojat et al. [47] find no differences in JSE results from students who answered online compared with those who answered in person. Further, 37.1% of the students in the sample declared that they did not live with their family, meaning that they most likely came from other provinces of the country. As described in other studies in Spain [28, 29] a greater number of women than men were found among the medical students. Considering the aforementioned observations, we believe that the sample of medical students from this research offers a fair representation of the medical degree student population at the national level.

There are no precise data in our setting that can explain the evolution of medical students’ empathy throughout the academic years. In this study, the academic year doesn´t decrease empathy. Indeed, it improved for men in their final academic year compared to their third year. Andersen et al. [56] conduct a systematic review of 30 studies carried out in 20 different countries over five continents (24 cross-sectional and 6 longitudinal studies) on the evolution of empathy in medical students over time; they report a deterioration of empathy in 14 studies. In the remaining 16, empathy improved or did not change, or the studies presented conflicting results. Cultural differences could be the cause of this heterogeneity of results, as evidenced by the systematic review [56] in which medical students from Asian countries scored lower in empathy than those from Western countries. A recent review by Ponnamperuma et al. [57] supports this idea, observing that while most US studies show a deterioration in empathy over time, other countries show a stabilization or even a modest increase. Other possible explanations for these differences could be the different designs (longitudinal or cross-sectional) or instruments used to measure empathy. A cohort study [31] conducted at one of the universities participating in this study centered on 102 medical students over a five-year period, showed that empathy did not decline during this time. In fact, it improved slightly in women, especially in terms of cognitive empathy.

To date, no studies have been published in Europe with a sufficiently broad and representative sample that would allow for a proposal of cut-points and tables with percentile ranks representing the population of a European country, which in turn would help in differentiating between students with high, medium, and low levels of empathy. This study offers, as do other authors in USA [46, 47], some cut-off points that can help us better analyze the levels of empathy of any first-year student enrolled in medical colleges in Europe at the beginning of the academic year, observe their evolution over time, and determine the impact of curricular activities that seek to improve them. As a result, we can prepare and train future physicians to have better clinical and human skills, as well as be more satisfied with their professional lives. Some authors argue that the teaching of all components of empathy, not only cognitive and behavioral aspects but also emotional and moral aspects, must be included in the curriculum of a degree in medicine. Different systematic reviews [58] support this thesis. For example, a man who begins his medical studies at a school of medicine and obtains a JSE score of 95 will find himself in the 11th percentile, and his level of empathy may be considered moderate. However, the same score in a woman would place her in the 3rd percentile, which corresponds with a low level of empathy. A medical school in another Spanish locality with sociodemographic data and JSE scores comparable to those presented in Table 2 could make use of the same cut-off points.

If we compare the cut-off points established by Hojat et al. in 2,637 medical students at Sidney Kimmel Medical College with those proposed in this study, we find that, except at the lower limit of empathy in men, other points that define empathy as high and low are higher in our study. We believe that this is a reflection of the higher average levels of global empathy found in Spanish medical students [30, 31], compared with those in US students [39, 46]. The empathy scores of medical students in this study showed moderate negative asymmetry (-0.95 in men and -0.94 in women). This means that the score curve moves to the right toward higher scores [59]. On another note, the curve is slightly leptokurtic [59] as the kurtosis values are positive (0.21 in men and 1.08 in women). These results are similar to those reported in other studies [46].

Our study confirms, as in previous studies [18, 46, 47, 50, 56], that women obtain higher empathy scores than men, although the magnitude of this difference is moderate. Different factors, such as social learning or genetic predisposition, could justify this finding [4].

Regarding MSWBI, 43.4% of women and 36.7% of men scored ≥4 points. Of concern is the high percentage of medical students with a high degree of psychological distress in the present study, although this percentage is less than the 52.9% recently reported by Rajapuram et al. [60] for medical students in the United States. Both studies were conducted prior to the start of the COVID-19 pandemic. Compared with men, women showed higher levels of empathy and were more involved in volunteer work. However, they also reported higher levels of psychological distress. A systematic review in the United States and Canada by Dyrbye et al. [41] finds higher levels of anxiety, depression, and stress in medical students in North America, compared with those reported by peers of the same age in the general population. In addition, the study found an association between psychological distress and a deterioration in academic performance, professionalism, and empathy toward patients.

In a systematic review, Wilkinson et al. [61] observed an inverse relationship between burnout and empathy. There have been many explanations for the cause of empathy deterioration suffered by medical students, although there is no clear and objective causal relationship. Such causal factors include stress [62], work overload, burnout [15, 63], contact with patients, lack of adequate mentors, hidden curricula, highly biologicist teaching, climate of cynicism, and distancing from the suffering of others [4, 64]. Identifying students with a high degree of psychological distress could prevent some of the fatal consequences described previously. Some medical schools [65] already offer programs to their students to improve their level of well-being, although such initiatives are still underdeveloped and their effectiveness has been poorly evaluated [66].

In line with what has already been purported by previous authors [50, 67–69], the present study finds that students who participate in volunteer work and choose people-centered specialities (general and medical) obtain better empathy scores than those who lean more toward technology, surgical procedures, and non-medical specialities. Guilera et al. [70], in a study of a single faculty of medicine in Spain, find an association between empathy and a preference for person-centered specialties in 110 students. This fact has also been observed [67] in medical students at the beginning of their first year of studies before the formal teaching commenced. Students with more empathy tended to choose specialties that require more frequent and intense contact with patients, such as internal medicine, pediatrics, family medicine, and psychiatry [50, 67–69, 71, 72]. The magnitude of this effect became clearer as the students chose specialities without direct treatment. This paper provides some relevant findings regarding this phenomenon, as in the previous systematic reviews that analysed it [56], only one-third find a clear association between empathy and medical specialties.

Awareness of the level of empathy in medical students and its determinants may enable us to identify students who most need concrete interventions to help reinforce the quality of empathy [4, 73]. Furthermore, it may help medical schools strengthen their curricula and offer better guidance to students in choosing a future specialty. Evaluating the degree of empathy in applicants of a medical degree may be a factor to consider during candidate selection, given the vital importance of this quality in medical practice [4]. In this way, we can train future doctors to maintain quality, humane, and meaningful relationships with their patients, which are mutually beneficial to both parties.

### Limitations

This research consists of a cross-sectional study with empathy analysis at three points in the medical students’ studies (their first, third, and sixth years). Therefore, we analyze different student samples, and not the same group, over time.

The present study evaluates empathy using a questionnaire that is self-administered and is not from the perspective of third parties (real or simulated patients or expert evaluations); nevertheless, some previous studies [19, 74–76] have demonstrated the correlation between these two methods of measurement.

The bias of social desirability may also have affected the results. Even so, Hojat et al. [47], in a study with students of osteopathic medicine, find that only 2.5% of the students were likely to give false “good impressions,” as detected by the Scale of the Zuckerman-Kuhlman Personality Questionnaire [77]. A recently published cohort study [31] finds no significant differences between the empathy levels of students who identified themselves compared with those who preferred to remain anonymous when answering the JSE. The large sample size of our research and the anonymous and voluntary nature of the questionnaire minimized the possible bias of social desirability.

### Future research

More studies with students from other medical schools in Spain are needed to confirm that the medical student populations all over the country are similar to those in Madrid. If this proves to be the case, the use of the same percentile tables and cut-off points proposed here may be justified throughout the country. The empathy of the students in this study should continue to be monitored longitudinally at least at three critical moments during their degree studies (first, third, and sixth years). It would also be interesting to measure students’ empathy from the perspective of patients (real or simulated) or communication experts and to analyze whether there is a relationship between their scores and the levels of empathy attained in this study. It is also desirable to relate the proposed levels of empathy to other variables [18–20] which are known to influence student empathy, such as their clinical performance or their best scores on the Objective Structured Clinical Examination. In this way, we can confirm the predictive validity of the cut-off points presented here.

This study found a relationship between the personal well-being of medical students and their levels of empathy. A systematic review of residents found that the higher the students’ well-being, the better their empathic ability [78]. Future studies are needed to analyze the determinants of students’ well-being throughout their degree studies, as well as their influence on their level of empathy.

It is necessary to evaluate the evolution of student empathy over time through longitudinal studies and to study the factors that determine students’ levels of empathy. This knowledge will help us implement curricular activities that encourage empathy among medical students. The EMMA project will present soon the influence of different academic study programs of all the medical schools in Madrid on the empathy levels of their students.

## Conclusions

The empathy of medical students in Madrid did not differ among the three critical moments of their university studies. The established cut-off points will allow identifying students with lower levels of empathy in populations similar to the one described here. This identification is essential for the subsequent implementation of curricular activities that aim to reinforce and enhance this quality to help promote a more person-centered and meaningful physician-patient relationship. Further, we observed a high percentage of medical students with considerable psychological distress. Therefore, an analysis of the causes is important to implement activities or structural changes that prevent the severe risks associated with such distress, such as anxiety, depression, suicidal ideation, and abandonment of medical studies.
